# A Unified Approach for EIT Imaging of Regional Overdistension and Atelectasis in Acute Lung Injury

**DOI:** 10.1109/TMI.2012.2183641

**Published:** 2012-01-10

**Authors:** Camille Gómez-Laberge, John H. Arnold, Gerhard K. Wolf

**Affiliations:** Harvard Medical SchoolDepartment of AnesthesiologyPerioperative and Pain MedicineChildren’s Hospital Boston Boston MA USA 02115

**Keywords:** Acute respiratory distress syndrome, electrical impedance tomography, lung imaging

## Abstract

Patients with acute lung injury or acute respiratory distress syndrome (ALI/ARDS) are vulnerable to ventilator-induced lung injury. Although this syndrome affects the lung heterogeneously, mechanical ventilation is not guided by regional indicators of potential lung injury. We used electrical impedance tomography (EIT) to estimate the extent of regional lung overdistension and atelectasis during mechanical ventilation. Techniques for tidal breath detection, lung identification, and regional compliance estimation were combined with the Graz consensus on EIT lung imaging (GREIT) algorithm. Nine ALI/ARDS patients were monitored during stepwise increases and decreases in airway pressure. Our method detected individual breaths with 96.0% sensitivity and 97.6% specificity. The duration and volume of tidal breaths erred on average by 0.2 s and 5%, respectively. Respiratory system compliance from EIT and ventilator measurements had a correlation coefficient of 0.80. Stepwise increases in pressure could reverse atelectasis in 17% of the lung. At the highest pressures, 73% of the lung became overdistended. During stepwise decreases in pressure, previously-atelectatic regions remained open at sub-baseline pressures. We recommend that the proposed approach be used in collaborative research of EIT-guided ventilation strategies for ALI/ARDS.

## I. Introduction

Mechanical ventilation is an essential part of caring for critically-ill patients with acute lung injury (ALI) or its more severe form acute respiratory distress syndrome (ARDS). Unfortunately, it has become clear after two decades of research that mechanical ventilation itself can exacerbate lung injury and, thus, increase the chance of death [1], [2]. Multicenter trials attempting to reduce the mortality among ALI/ARDS patients by strategic selection of ventilation parameters have led to mixed results [3]–[6]. Their goal was to find a lung protective tidal volume ($V_{\rm T}$) and positive end-expiratory pressure (PEEP) according to measurements that characterize the lung as a whole. Advances in lung imaging research are now calling into question the presumption that whole-lung measurements are sufficient for identifying lung protective ventilation settings for ALI/ARDS. Computed tomography (CT) and, more recently, electrical impedance tomography (EIT) show that the mechanical properties of the ALI/ARDS lung are, in fact, very heterogeneous [7], [8]. Furthermore, the percentage of collapsed, but potentially recruitable, lung throughout the patient population is highly variable [9]. In some patients, typical ventilator settings can lead to regional lung overdistension and atelectasis, which correlate with subsequent lung injury [10], [11]. Consistent with these findings, a meta-analysis combining the data from multicenter trials has shown that only the *subgroup* of patients with ARDS showed a reduction in mortality to higher levels of PEEP [12]. Everything considered, ALI/ARDS affects lung mechanics in a heterogeneous manner. Therefore, a reduction in mortality among patients will most likely require a ventilation strategy based on regional indicators of lung mechanics.

In this paper, we consolidate various techniques from recent electrical impedance tomography (EIT) research into a unified method for imaging regional lung overdistension and atelectasis during mechanical ventilation of the ALI/ARDS patient. Our objective is to encourage multicenter collaboration by proposing a comprehensive software solution for all data processing stages: from data acquisition to physiological interpretation. The design criteria we have selected for this purpose are: *currency* of the techniques used, *compatibility* with most EIT systems, *transparency* of the software implementation, physiological *interpretability* of output, and *automaticity* of processing where possible. We implement and demonstrate a software solution according to these criteria, which is available to all investigators, and amenable to updates and improvements. We believe that the output from an EIT system utilizing such software would be physiologically interpretable by clinical staff and directly comparable with output from other centers.

The solution is implemented on the software platform of the Electrical Impedance and Diffuse Optical Reconstruction Software (EIDORS) project.^1^^1^Available at http://www.eidors.org EIDORS is maintained on the Internet by EIT researchers worldwide; it is platform-independent and compatible with most EIT systems. The data acquisition and image reconstruction algorithms used here conform to the Graz consensus on EIT lung imaging (GREIT), as described by Adler et al. [13]. GREIT provides the broadest consensus on EIT lung imaging techniques for electrode placement, stimulation and measurement patterns, as well as image reconstruction algorithms. Upon this framework, we develop an automatic filtering technique to attenuate nonrespiratory signals and to detect peak inspiratory and expiratory time points for the measurement of regional tidal volume. The corresponding filtered images are then used for lung region classification based on the functional EIT (fEIT) method of Pulletz et al. [14]. We embed the fEIT method within an algorithm to automatically determine the threshold parameter for lung classification and to aggregate lung regions identified over time. The tidal volume images and ventilator pressure measurements are combined into maps of regional respiratory system compliance.^2^^2^Respiratory system compliance is equal to lung compliance plus chest wall compliance. We represent temporal changes in respiratory system compliance with a pair of images: the first quantifies the maximum compliance at each pixel, and the second indicates the ventilatory pressure when it was achieved. These results are used to estimate the percentage and location of lung overdistension and atelectasis using the method of Costa et al. [15].

We evaluate the method’s performance using EIT data acquired from patients with ALI/ARDS who underwent a lung recruitment maneuver followed by a PEEP titration strategy within our pediatric intensive care unit.

## II. Methods

### A. Lung Recruitment Data Set

The proposed methods were used to monitor lung mechanics in pediatric patients with ALI/ARDS participating in a clinical study of lung recruitment (ClinicalTrials identifier: NCT00830284). The study protocol was approved by the hospital’s Institutional Review Board, and written informed consent was obtained from a parent or guardian prior to enrollment. At the time of enrollment, the patients met the ALI/ARDS criteria for less than 72 h [16]. Throughout the study, the patients were either deeply sedated demonstrating apnea or received neuromuscular blocking agents.

**Fig. 1.**
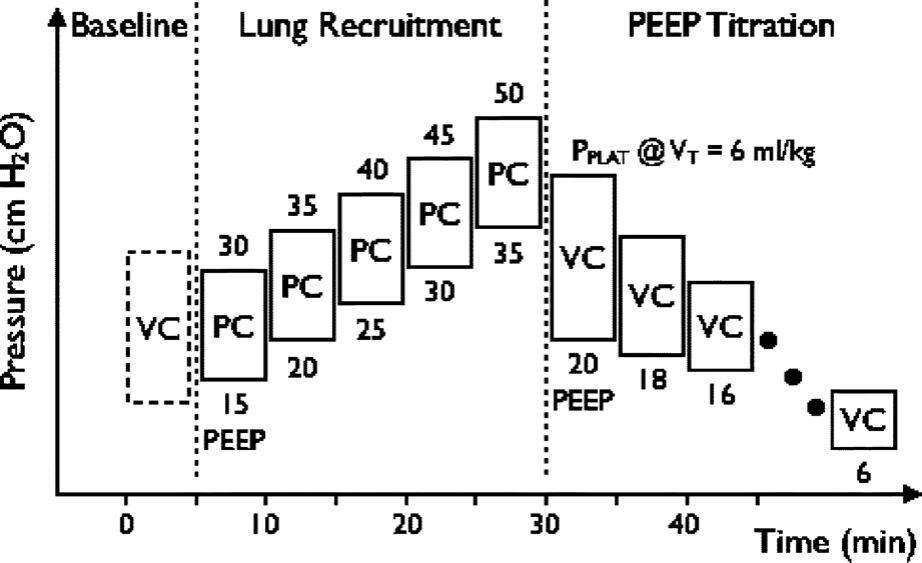
The experimental protocol is divided in three stages. Baseline PEEP was determined according to clinical practice. During the “baseline” and “PEEP titration” stages, patients were ventilated in VC mode with $V_{\rm T}=6.0 {\rm ml}/{\rm kg}$, and $\Delta P_{\rm awo}$ was measured by subtracting PEEP from the measured $P_{\rm PLAT}$. During the “lung recruitment” stage, patients were ventilated in PC mode, and $\Delta P_{\rm awo}$ was set to 15 cm ${\rm H}_{2}{\rm O}$ for each step. VC: volume-controlled; $P_{\rm PLAT}$: plateau pressure; PC: pressure-controlled.

The time course of the experimental protocol is divided into a baseline ventilation stage, a lung recruitment stage where airway pressure was increased sequentially, and a PEEP Titration stage where airway pressure was decreased sequentially (Fig. 1). During the baseline stage, patients were ventilated in a volume-controlled (VC) mode ($V_{\rm T}=6.0 {\rm ml}/{\rm kg}$), where PEEP was set according to clinical practice. We also measured the difference between the plateau pressure ($P_{\rm Plat}$) and PEEP at the airway opening

$$\Delta P_{\rm awo} = P_{\rm Plat} - {\rm PEEP}.\eqno{\hbox{(1)}}$$

During the lung recruitment stage, patients were switched to pressure-controlled (PC) ventilation ($\Delta P_{\rm awo} = 15 {\hbox {cm}} {\rm H}_{2}{\rm O}$), and PEEP was increased by 5 cm ${\rm H}_{2}{\rm O}$ with the objective of meeting a predefined blood gas criterion of lung recruitment [17]

$$P_{{\rm aO}_{2}} + P_{{\rm aCO}_{2}} \geq 400\, {\rm mm Hg} \eqno{\hbox{(2)}}$$

where $P_{{\rm aO}_{2}}$ and $P_{{\rm aCO}_{2}}$ are the partial pressures of oxygen and carbon dioxide in arterial blood, respectively. We used this recruitment index, since it was shown to consistently coincide with anatomical recruitment measured by CT in patients with early ALI/ARDS [17]. The lung recruitment stage was stopped before reaching the index if PEEP reached 35 cm ${\rm H}_{2}{\rm O}$, or if any of the following stopping criteria were met: arterial oxygen saturation $< 80\%$, ${\hbox {arterial pH}} < 7.00$, ${\hbox {arterial lactate}} \geq 2 {\rm mg}/{\rm dl}$, mean arterial blood pressure decreases by 20%, or the required dosage of vasoactive agents increases by 50%. During the PEEP Titration stage, patients were switched back to VC mode ($V_{\rm T}=6.0 {\rm ml}/{\rm kg}$), and PEEP was decreased to the lowest possible setting that satisfied (2). In our approach, PEEP was initially set at 20 cm ${\rm H}_{2}{\rm O}$ and was decreased in steps of 2 cm ${\rm H}_{2}{\rm O}$ until one of the following: the minimum PEEP satisfying (2) was found, ${\rm PEEP} =6 {\hbox {cm}} {\rm H}_{2}{\rm O}$, or if any of the aforementioned stopping criteria were met.

### B. Data Acquisition and Lung Imaging

EIT measurements were performed using either the Goe-MF II (CareFusion, San Diego, CA) or the Dräger EIT Evaluation KIT 2 (Dräger Medical, Lübeck, Germany), based upon availability of the devices in the intensive care unit. Both devices are designed to operate with similar specifications for the purpose of single frequency EIT. Stimulation current, electrode impedances and voltages are comparable in both devices. The image reconstruction and processing routines proposed here were applied to the raw voltage data stream from both systems.

#### 1. Electrode Placement

Sixteen coplanar electrodes were placed equidistantly around the thorax at the level of the parasternal sixth intercostal space. The reference electrode was placed on the right side of the abdomen near the waistline. Electrodes #1 and #16 were symmetrically placed to the left and the right of the sternum, respectively, so that electrodes #8 and #9 straddled the spinal column. This configuration leads to transverse images in the radiological convention. Each image was reconstructed from voltage measurements acquired with *adjacent current stimulation* [13] (50 kHz, 5 mA rms), at a frame rate of 20 Hz. To maintain good signal quality, we ensured that electrode–skin resistance did not exceed 200 $\Omega$ at any time.

#### 2. Image Reconstruction

Image reconstruction was performed using the EIDORS implementation of the *one-step linear Gauss–Newton solver* with a regularization term for conductivity change and another accounting for electrode movement [13]. We calculated changes in regional impedance according to the *normalized difference* between electrode voltages at each time point $V$ and a reference $V_{\rm ref}$, which was obtained by averaging voltage data corresponding to peak-expiration. We chose this reference because it would represent all impedance changes as positive quantities for all lung volumes. Thus, for each measurement, we have the nonnegative quantity $y = (V - V_{\rm ref})/V_{\rm ref}$.

In the GREIT notation, changes in all of the $n_{M}$ voltage measurements and $n_{E}$ electrode positions are written in vector form ${\bf y} \in \BBR ^{n_{M}+ n_{E}}$. These can be calculated by solving the forward problem, where ${\bf y}$ is obtained from a linear operation on the conductivity distribution ${\bf x} \in \BBR ^{n_{N}}$ over the $n_{N}$ regional elements representing the thorax. The linear model is

$${\bf y} = {\bf J} {\bf x} + {\bf n} \eqno{\hbox{(3)}}$$

where ${\bf J} \in \BBR ^{n_{M} \times (n_{N} + 2 n_{E})}$ is the Jacobian matrix for changes in conductivity and electrode position [18], and ${\bf n} \in \BBR ^{n_{M} + n_{E}}$ is additive white noise. Each element of ${\bf x}$ quantifies the change in regional conductivity. In later sections on physiological interpretation, we will use the reciprocal of ${\bf x}$, called the regional impedance $\Delta Z$, since it is more commonly used in the clinical literature on EIT. Furthermore, $\Delta Z$ has been shown to correlate with changes in regional tidal volume [19]–[21].

The estimate of ${\bf x}$ is obtained by solving the inverse problem as follows [22]:

$${\mathhat {\bf x}} = ({\bf J} ^{t} {\bf J} + \lambda ^{2} {\bf R})^{-1} {\bf J} ^{t} {\bf y} \eqno{\hbox{(4)}}$$

where $t$ indicates the matrix transpose, and $\lambda$ and ${\bf R}$ are the regularization parameter and matrix, respectively. We tuned $\lambda$ so that the amount of noise amplification remained constant between patient data sets. This was done for each data set by setting the *noise figure* to 0.60 [23].

The finite element models used for the forward and inverse problems are labeled *d2t2* and *d2t3*, respectively, in the EIDORS project. They are 2-D meshes with point electrodes that approximate the contour of the human thorax passing through the electrode plane.

We reconstructed an image as described above for every time point during EIT scanning in order to obtain a image series during mechanical ventilation. The following methods pertain to calculations using the image series acquired during each step of the experimental protocol.

#### 3. Intra-Tidal Impedance Changes

The image series was used to detect the time points of peak-inspiration and peak-expiration. An average time signal was computed by averaging all pixels within each image. The time signal was bandpass-filtered in order to isolate the respiratory component. The lower corner frequency (attenuating the signal by 3 dB) was $f_{\rm lower} = 0.01 {\rm Hz}$. The choice for the upper corner frequency $f_{\rm upper}$ was determined automatically, since it depends on the respiratory rate. We used the magnitude of the Fourier-transformed signal to identify the frequency with the largest spectral peak $f_{\rm peak}$ greater than $f_{\rm lower}$ and set $f_{\rm upper} = 2f_{\rm peak} {\rm Hz}$.

We used the implementation of a 128-point zero-phase finite impulse response filter by calling the function *filtfilt* from the Matlab Signal Processing Toolbox. The rationale for using a zero-phase filtering technique was to preserve the accurate peak-inspiration and peak-expiration times by canceling the linear-phase shift associated with digital filters. These time points were used to estimate regional ventilation by calculating the average difference in $\Delta Z$ between peak-inspiration and peak-expiration. Note that $\Delta Z$ is insensitive to aerated but nonventilated lung regions.

#### 4. Lung Region of Interest Detection

The lung region of interest (ROI) was determined by applying a threshold to the *functional EIT* image (fEIT) computed from an image series during tidal ventilation [14]. The pixel intensity of the fEIT image represents the standard deviation of $\Delta Z$ during tidal breathing in the bandpass-filtered image series. Pixels are considered part of the lung ROI if they surpass a threshold value $0 \leq \theta \leq 1$ representing a percentage of the maximum standard deviation in the fEIT image. For each image series, we determined the value for $\theta$ automatically by searching for the ROI with the smallest number of inconsistently classified pixels when compared to ROI obtained for $\theta -0.025$ and $\theta + 0.025$. The ROI templates were then merged by the logical “or” operation, yielding the aggregate ROI, that we used for calculating regional respiratory system compliance, atelectasis, and overdistension.

**Fig. 2.**
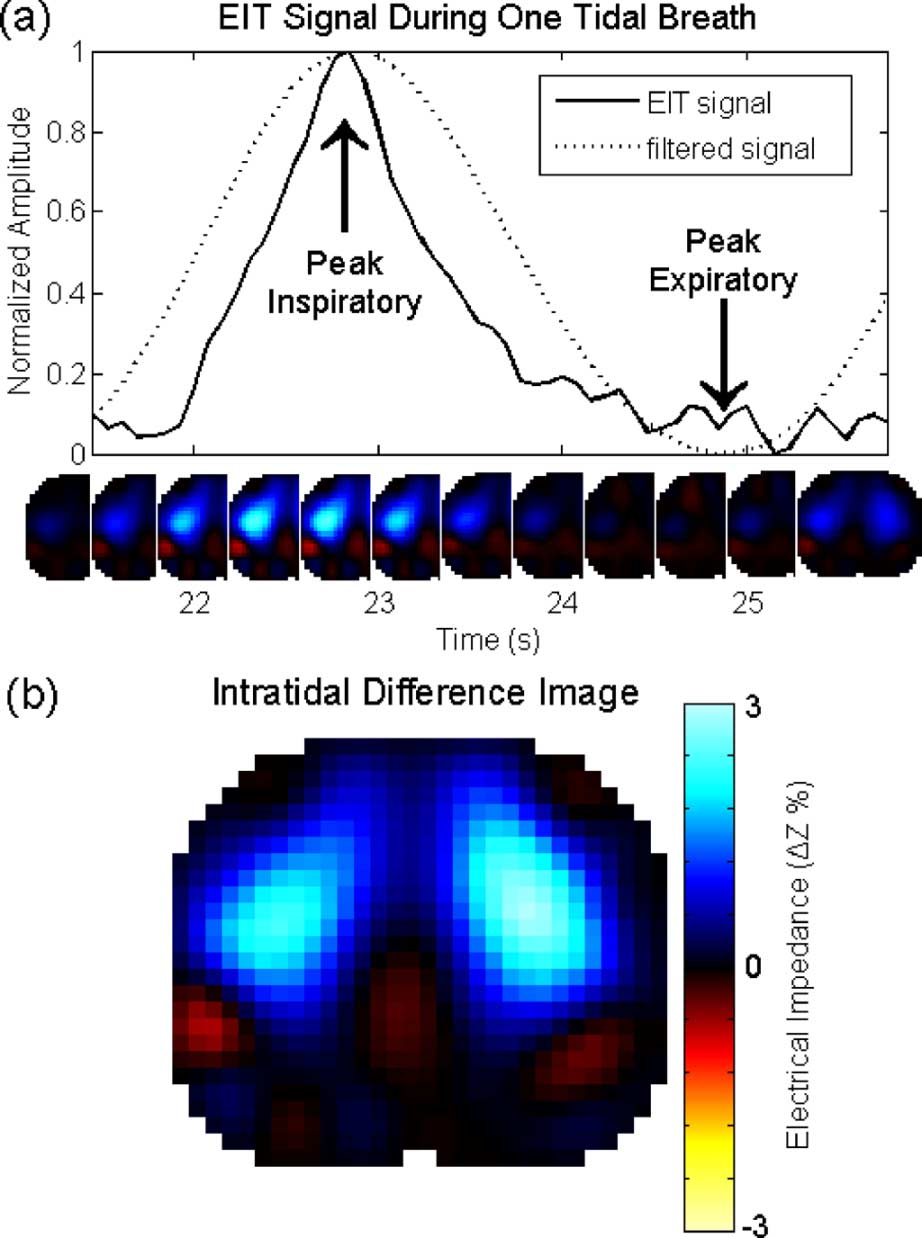
Respiratory frequency bandpass-filtered EIT image series captured during one tidal breath. (a) Graphs of the original and bandpass-filtered EIT signals plotted versus time. The extrema of the filtered signal is utilized to identify peak inspiratory and expiratory time points. (b) Corresponding intra-tidal difference image, a correlate of regional lung ventilation, obtained by subtracting the peak expiratory image from the peak inspiratory image. The colormap applies to all images and represents increases (cool colors) and decreases (warm colors) relative to an average peak-expiratory reference image. Images represent the transverse view in the radiological convention (ventral: image top; right side: image left).

#### 5. Dynamic Respiratory System Compliance Estimation

Changes in dynamic respiratory system compliance were estimated for each pixel in the EIT image by the ratio

$$C_{\rm dyn} = {{\Delta Z}\over {\Delta P_{\rm awo}}}.\eqno{\hbox{(5)}}$$

We used changes in $C_{\rm dyn}$ as a function of PEEP to estimate the magnitude of overdistension and atelectasis [15]. During each stage of the protocol, dynamic compliance was computed for each PEEP setting $P$, and we determined each pixel’s maximum compliance and the PEEP setting when it was achieved

$$\eqalignno{C_{\rm max} =&\, \max \{C_{\rm dyn}(P)\} & \hbox{(6)}\cr P^{\ast } =&\, \arg \max \{C_{\rm dyn}(P)\} & \hbox{(7)}}$$

where $\arg \max$ returns the set of pressures $P^{\ast }$ that maximize the compliance signal for each pixel in the lung ROI.

#### 6. Regional Overdistension and Reversible Atelectasis Estimation

The fraction of overdistended or atelectatic units in a given pixel at PEEP setting $P$ can be obtained in terms of $C_{\rm max}$ and $P^{\ast }$ as follows [15]:

$$F(P) = \cases{\hfill {{C_{\rm dyn}(P)-C_{\rm max}}\over {C_{\rm max}}}, & if $P < P^{\ast }$\hfill \cr \hfill {{C_{\rm max}-C_{\rm dyn}(P)}\over {C_{\rm max}}}, & if $P\geq P^{\ast }$.\hfill } \eqno{\hbox{(8)}}$$

This function is bounded between $[{-1,1}]$. For pressures above $P^{\ast }$, a pixel within the lung ROI represents lung tissue that is $F(P) \times 100\%$ overdistended. For pressures below $P^{\ast }$, the corresponding lung tissue is $-F(P)\times 100\%$ atelectatic. We apply (8) to the EIT image pixel data acquired for every step of the protocol. We present our data in two formats: as images representing the regional distribution of lung overdistension and reversible atelectasis for each patient, and as graphs aggregating all patient data as a $C_{\rm max}$-weighted pixel average that is plotted versus $P$ throughout the entire lung ROI.

**Fig. 3.**
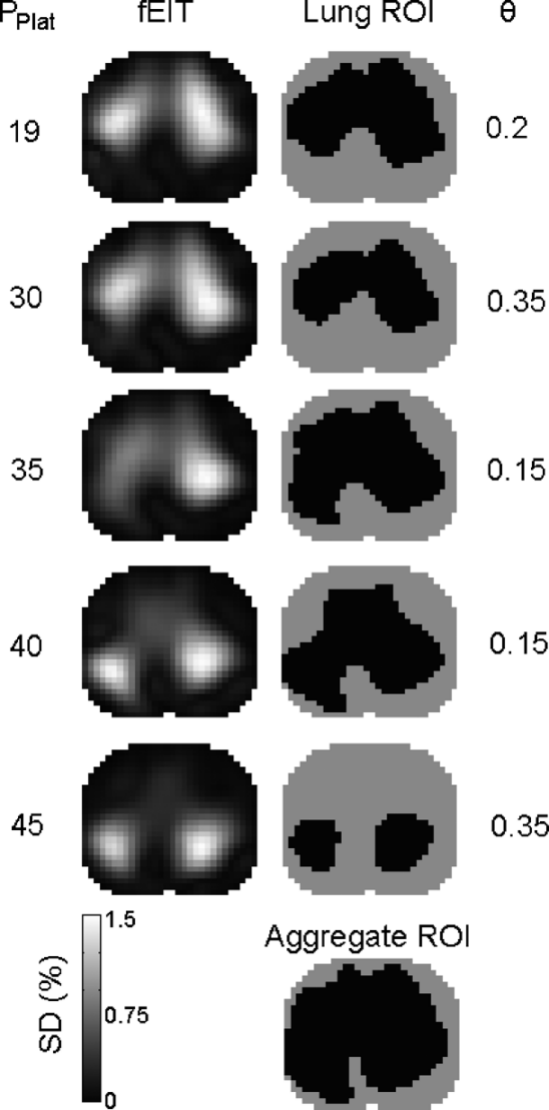
Aggregation of the lung ROI template during the baseline (top row) and lung recruitment stages. The fEIT images from the filtered image series (left column) demonstrate how the signal locus shifts from ventral to dorsal lung regions. The corresponding ROI templates (right column) represent nonlung (gray) and lung (black) regions, where the standard deviation (SD) threshold $\theta$ was determined automatically. The aggregate ROI (bottom right) is obtained by applying the logical “or” operation on all ROI templates (black represents “true” and gray “false”).

## III. Results

### A. EIT Images of Intra-Tidal Impedance Change

Isolation of the respiratory signal with zero-phase filtering led to the accurate detection of peak inspiratory and expiratory time points. For each step of the protocol, 15 complete breaths were recorded on average. The total number of breaths recorded in all patients was 1087, of which 1044 (96.0%) were detected and 43 (4.0%) were missed. There were also 27 (2.4%) false detections, where we counted each inter-breath period as a true negative. Therefore, the sensitivity and specificity of the breath detection algorithm was 96.0% and 97.6%, respectively. Incorrect detections were either manually adjusted or discarded for further analysis. The filtered image series during a typical tidal breath is shown along with its corresponding intra-tidal difference image (Fig. 2). The intra-tidal difference image estimates the regional tidal volume and is obtained by subtracting the peak expiratory image from the peak inspiratory image. We estimated the error in intra-tidal timing and regional volume by comparing the automatically detected time points against those identified manually from the raw EIT signal. From each patient, we calculated the error $t_{\rm manual} - t_{\rm auto}$ for peak-inspiratory and peak-expiratory time points from five randomly-selected breaths. From these breaths, we also calculated the error in estimated tidal volume $(\Delta Z_{\rm manual}-\Delta Z_{\rm auto})/\Delta Z_{\rm manual}$. The error for peak-inspiratory and peak-expiratory time point detection was $-0.02\pm 0.15 {\rm s}$ and $0.21\pm 0.41 {\rm s}$, respectively (${\rm mean} \pm {\rm SD}$, $n=45$). Neither were significantly different from zero (paired $t$-test, $p>0.05$). The larger error of the peak-expiratory time point was expected, since the expiratory phase of the signal is flat for at least 1/2 s; therefore, the time point of the minimum is susceptible to noise [Fig. 2(a)]. These timing errors led to the underestimation of tidal volume by $5.4\pm 16.8\%$ (${\rm mean} \pm {\rm SD}$, $n=45$).

The intra-tidal impedance changes were detected after bandpass filtering. Throughout the protocol, in all patients, the respiratory component was adequately isolated. The mean and its standard error for the respiratory rate throughout the protocol was $23.5 \pm 2.7$ breaths/min ($0.392 \pm 0.045 {\rm Hz}$). The heart rate was $109 \pm 15$ beats/min ($1.8 \pm 0.25 {\rm Hz}$). The filter corner frequencies were $f_{\rm lower}=0.01 {\rm Hz}$ and $f_{\rm upper}=0.811 \pm 0.107 {\rm Hz}$, respectively. Hence, on average, the respiratory signal was well centered in the passband, and the cardiac signal was attenuated by more than 3 dB.

The fEIT image was computed on the bandpass-filtered image series in order to isolate the respiratory signal before lung ROI detection. A fEIT image for each step of the protocol, and the aggregate lung ROI was then obtained by performing the logical “OR” on all step-specific ROI (Fig. 3). The parameter $0 \leq \theta \leq 1$ represents the threshold of standard deviation relative to the image maximum, above which pixels are included in the lung ROI. The recommended range for manual ROI selection is $0.20 \leq \theta \leq 0.35$, [14]. The automatically-determined threshold had a mean of 0.24 and median 0.20 and, thus, was in agreement with manually selected lung ROI. Of the 73 set of data collected from all patients (one set per protocol step), 32 (44%) were below the manual range, 27 (37%) were within the range, and 14 (19%) were above the range.

### B. Regional and Total Respiratory System Compliance

**Fig. 4.**
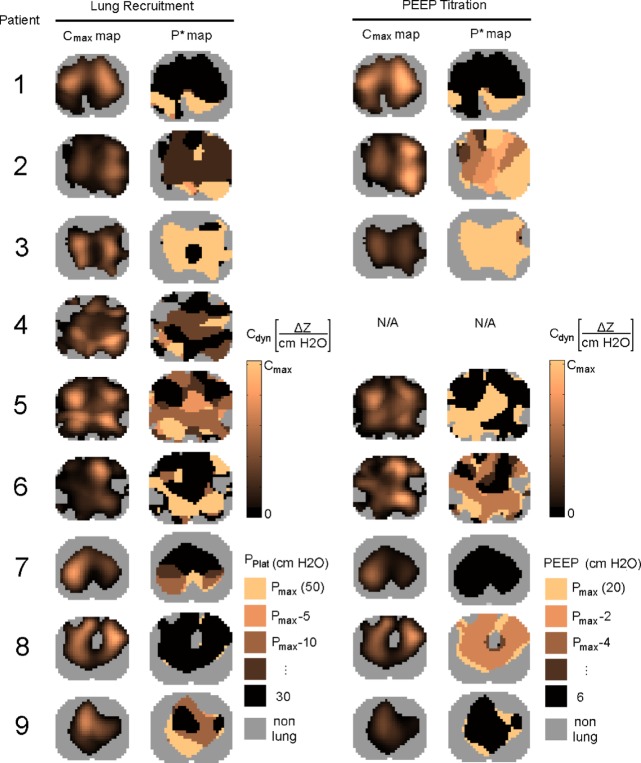
Maximum regional respiratory system compliance ($C_{\rm max}$ maps) and the corresponding ventilatory pressure ($P^{\ast }$ maps) are shown for lung recruitment and PEEP Titration stages of each patient. The $C_{\rm max}$ maps use a scale relative to each patient’s maximum compliance achieved throughout the entire protocol. The $P^{\ast }$ maps represent the pressure at which each pixel’s maximum compliance was achieved. For the recruitment stage, $P^{\ast }$ represents $P_{\rm Plat}$, and for the titration stage, $P^{\ast }$ represents PEEP. Bright colors represent maxima achieved at high pressures, while dark colors represent maxima achieved at low pressures.

For each patient, we present the changes in regional respiratory system compliance separately for the lung recruitment and PEEP Titration stages. In order to display the maximum regional compliance at every pixel and the pressure at which this compliance was achieved, we use a pair of images labeled $C_{\rm max}$ and $P^{\ast }$ whose pixel values correspond to the quantities satisfying (6) and (7), respectively (Fig. 4). Each row of Fig. 4 shows the regional compliance data for one patient. The first pair of images shows the maximum regional compliance at each pixel (i.e., $C_{\rm max}$ map) and the corresponding plateau pressure $P_{\rm Plat}$ needed for each pixel to reach its maximum (i.e., $P^{\ast }$ map) during lung recruitment. The second pair of images shows the $C_{\rm max}$ and $P^{\ast }$ maps in the same format for the PEEP Titration stage with the exception that the $P^{\ast }$ map quantifies PEEP. Because the maximum respiratory system compliance achieved by each patient was highly variable, ranging from 0.1–0.9 $\Delta Z/({\hbox {cm}} {\rm H}_{2}{\rm O})$, the $C_{\rm max}$ color scale was set according to the individual. Regions of high compliance are bright, while those of low compliance are dark. For each patient, the same color scale was used for both stages. The $P^{\ast }$ map uses dark colors to show regions that reached maximum compliance at low pressures, and bright colors for regions that reached maximum compliance at high pressures. Again, because each patient ended either stage at different pressures, the $P^{\ast }$ map color scale was set according to the individual. Patient #4 met safety-related stopping criteria during lung recruitment and, therefore, did not undergo PEEP titration. These results suggest that a considerable degree of heterogeneity exists throughout the lungs of each patient. All $C_{\rm max}$ maps contain a patchy distribution of regions of low and high compliance. In most cases, compliance throughout the whole lung is not maximized at a single pressure as shown by the mosaic pattern of the $P^{\ast }$ maps. This heterogeneity of compliance does not change significantly during the protocol, since the maps obtained from the lung recruitment and PEEP Titration stages are very similar. There is virtually no difference in the lung ROI between stages, and the locations of high compliance also do not change remarkably. A comparison of the $P^{\ast }$ maps for each patient shows that the dorsal region compliance values reached during the lung recruitment stage are maintained at much lower pressures during PEEP Titration stage. Similar results exhibiting this form of lung volume hysteresis have been shown in spirometry studies of the ARDS lung [24].

**Table I:** ${\rm Mean} \pm {\rm SD}$ of Pressure and Compliance Data. R, T, and Lung % Denote Lung Recruitment, PEEP Titration, and Fraction of Lung at Its Maximum Regional Compliance

*Protocol Step*	*P*_Plat_ (cm H_2_O)	PEEP (cm H_2_O)	*C*_max_	
			(${{\Delta Z}\over{{\rm{cm}}\,{\rm{H}}_2 {\rm{O}}}}$)	(*Lung* %)
Baseline	27 ±6	16 ±3	0.3 ±0.3	52 ±36
R1	30	15	0.2 ±0.3	24 ±21
R2	35	20	0.1 ±0.09	21 ±18
R3	40	25	0.1 ±0.06	3±6
R4	45	30	0.1 ±0.09	9±9
R5	50	35	0.1 ±0.3	15 ±23
T1	34 ±8	20	0.3 ±0.3	38 ±37
T2	28 ±4	18	0.3 ±0.2	31 ±34
T3	23 ±3	16	0.4 ±0.2	19 ±23
T4	22 ±2	14	0.5 ±0.2	46 ±19
T5	21 ±1	12	0.3 ±0.2	28 ±24
T6	20 ±1	10	0.3 ±0.1	12 ±8
T7	17 ±1	8	0.2 ±0.1	3±2
T8	16 ±1	6	0.1 ±0.02	1±0.3

In order to examine how the lung mechanics evolved during the lung recruitment and PEEP Titration stages, we pooled the pressure and compliance data from all patients for each step of the protocol (Table I). The fourth column of Table I shows the $C_{\rm max}$ average over the entire lung ROI. As a result of increasing plateau pressure by 23 cm ${\rm H}_{2}{\rm O}$, the respiratory system compliance dropped from a baseline value of 0.3 $\Delta Z/({\hbox {cm}} {\rm H}_{2}{\rm O})$ to 0.1 $\Delta Z/({\hbox {cm}} {\rm H}_{2}{\rm O})$ during the lung recruitment stage. Upon transition to the first step of the PEEP Titration stage (“T1” in Table I), the effects of hysteresis were already apparent. Respiratory system compliance returned to the baseline level at 0.3 $\Delta Z/({\hbox {cm}} {\rm H}_{2}{\rm O})$, effectively tripling the compliance measured at comparable pressure during step “R2” of lung recruitment. Reduction in pressure during PEEP Titration further increased compliance, which exceeded baseline and reached a maximum of 0.5 $\Delta Z/({\hbox {cm}} {\rm H}_{2}{\rm O})$ at step “T4.” Reduction in pressure below values in step “T4” appeared to reduce compliance to levels smaller than baseline. The last column of Table I shows the percentage of pixels within the lung that met their maximum compliance during each step of the protocol. While pressure increased during the lung recruitment stage, the fraction of pixels at their maximum compliance fell from 52% to as low as 3% at step “R3.” This effect was reversed during the PEEP Titration stage, where as much as 46% of pixels became maximally compliant at step “T4.” This extent of maximal regional compliance was not sustained with the lower pressures in steps “T5-T8.”

Since our ring of EIT electrodes were closest to the basal lung regions, we asked whether our measurement of compliance is representative of the whole lung in ALI/ARDS patients. We compared the changes in respiratory system compliance measured by EIT to those measured by the ventilator by calculating the Pearson product-moment correlation coefficient $r$ between the time series of measurements from each instrument (Table II). From our nine patients, we found that six had very strongly correlated signals that reached statistical significance ($0.82 \leq r \leq 0.96$, $p< 0.05$). In two other patients, the correlations were strong but only showed a trend toward significance ($r=0.67$, $p=0.07$, and $r=0.86$, $p=0.06$). In contrast, the EIT data from patient #8 were weakly correlated ($r=0.29$, $p=0.44$). While this case suggests that EIT was rather insensitive to changes in the gross lung, we cannot exclude the possibility that patient manipulations during the protocol may have inadvertently interfered with the EIT measurements.

### C. Regional Lung Overdistension and Atelectasis

**Fig. 5.**
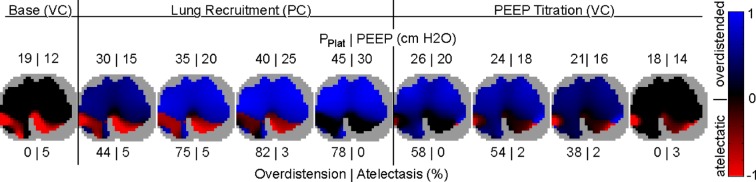
Regional overdistension and atelectasis maps in one patient throughout the protocol. Each map quantifies the degree and location of overdistended (blue) and atelectatic (red) lung units. Nonlung regions are shown in gray. VC: volume-controlled; PC: pressure-controlled.

The fraction of overdistended and atelectatic lung regions for each step of the protocol were obtained in terms of $C_{\rm max}$ and $P^{\ast }$ according to (8). The maps for regional overdistension and atelectasis are demonstrated for one patient in Fig. 5. The color of each pixel within the lung corresponds to the value $-1\leq F(P) \leq 1$ from (8). Each image answers the question of “how much” and “where” overdistension (blue) and atelectasis (red) takes place during each step of the protocol. Plateau pressure and PEEP are shown above each image, and pixel-averaged overdistension and atelectasis are shown below. In this patient, overdistension took place in as much as 82% of the lung ROI during the lung recruitment stage. The most severe overdistension was in the ventral lung regions. Interestingly, during the fourth and final step of lung recruitment, overdistension fell back to 78%, suggesting the aeration of novel regions. The effect of this regional opening can be seen as a retreat of the interface between ventral overdistension (blue pixels) and adequate aeration (black pixels). Before commencing the PEEP Titration stage, the nondependent lung is almost completely overdistended. The benefit of these high pressures can be seen in the reversal of atelectasis (red pixels) in the dependent lung. A $P_{\rm Plat}=40 {\hbox {cm}} {\rm H}_{2}{\rm O}$ was necessary to reverse atelectasis in 5% of the lung down to 3%. At the end of the protocol, atelectasis was confined to 3% of the lung utilizing $P_{\rm Plat}=18 {\hbox {cm}} {\rm H}_{2}{\rm O}$ and ${\rm PEEP}=14 {\hbox {cm}} {\rm H}_{2}{\rm O}$. This was less than half of the pressure required to achieve the same degree of atelectasis reversal during the lung recruitment stage.

**Fig. 6.**
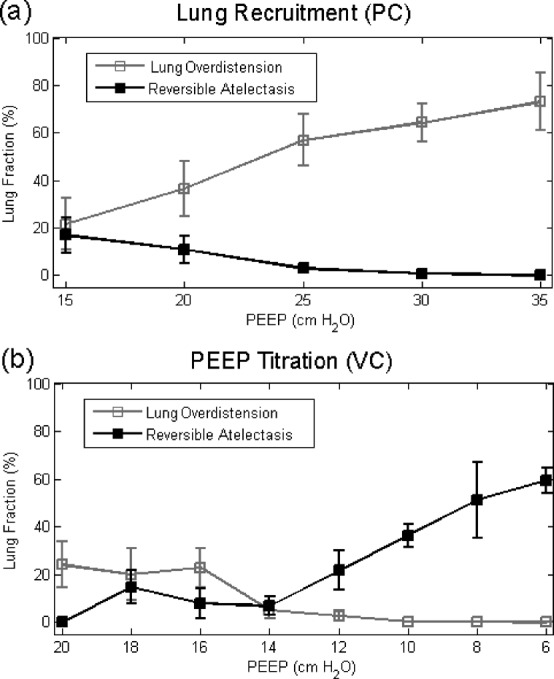
Lung overdistension (gray) and atelectasis (black) for all patients are plotted versus PEEP during (a) lung recruitment and (b) PEEP titration. For clarity, the bars represent the standard error. PC: pressure-controlled ventilation; VC: volume-controlled ventilation.

The mean and standard error of pixel-averaged overdistension and atelectasis for all patients are shown in aggregate during lung recruitment and PEEP Titration in Fig. 6. During the lung recruitment stage, the extent of lung overdistension increased monotonically from $21\pm 11\%$ to $73\pm 12\%$ with $P_{\rm Plat}$ climbing from 30 to 50 cm ${\rm H}_{2}{\rm O}$ [Fig. 6(a)]. This recruitment strategy resulted in the resolution of atelectasis in $17\pm 8\%$ of the lung [Fig. 6(a)]. During the PEEP Titration stage, while PEEP was reduced from 20 to 14 cm ${\rm H}_{2}{\rm O}$, overdistension was maintained within approximately 20% of the lung, and atelectasis was maintained within 10% of the lung [Fig. 6(b)]. The extent of overdistension and atelectasis was smaller on average during PEEP Titration (overdistension $23\pm 6\%$; atelectasis $10\pm 3\%$) than during lung recruitment (overdistension $30\pm 7\%$; atelectasis $15\pm 3\%$) when comparing these stages at equal pressures $15\leq {\rm PEEP}\leq 20 {\hbox {cm}} {\rm H}_{2}{\rm O}$ [compare Fig. 6(a) and (b)]. Reducing PEEP beyond 14 cm ${\rm H}_{2}{\rm O}$ led to a monotonic increase in atelectasis from $7\pm 4\%$ to a maximum of $59\pm 5\%$ [Fig. 6(b)].

In summary, stepwise increases in pressure could reverse atelectasis within 17% of the lung, appearing primarily in the dorsal regions. During the highest pressures applied, 73% of the lung became overdistended, appearing primarily in the ventral regions. Overdistension subsided during stepwise decreases in pressure, and previously-atelectatic regions remained open at pressures below those at baseline. Together, these results suggest that the described ventilation strategy can effectively recruit the lung, and that EIT imaging can be used to guide the reduction of ventilatory pressures to avoid re-collapse of the lung.

## IV. Discussion

Building upon a decade of experimental validation of EIT regional lung volume measurements [19], [25]–[29], there has been a recent surge in EIT research about mechanical ventilation of the ALI/ARDS lung [15], [30]–[35]. These studies provide important insights into how the EIT data stream can be interpreted physiologically. Most recently, the following methods have been proposed: an intra-tidal respiratory system compliance index [15], fEIT changes between PEEP settings [30], a respiratory system compliance percentile change from a scaled baseline value [31], a regional lung state classification index based on changes between PEEP settings [32], the dorsoventral center-of-mass coordinate of a spectral power EIT image [33], an intra-tidal global ventilation inhomogeneity index [34], and ventilator-calibrated regional distributions of end-expiratory lung volume and potentially-recruitable lung volume [35]. In addition to this variety of proposed indexes of lung mechanics, each assumes that a reconstruction algorithm has already computed the images from the voltage measurements. However, it is difficult to compare results obtained from arbitrary reconstruction algorithms, since the ill-posed nature of the EIT conductivity problem can cause different algorithms to generate rather divergent images from the same data [36].

In this paper, we combined various EIT techniques to form a complete sequence for interactively quantifying regional lung overdistension and atelectasis from EIT voltage and airway pressure data. The methods were chosen according to criteria that we deemed essential for developing EIT-guided mechanical ventilation within the critical care setting. The lung imaging methods conform to the GREIT specifications [13], which are recognized by a large collaborative network of scientific and clinical experts as being the state-of-the-art in EIT lung imaging. Image reconstruction was essentially automatic, since only the regularization parameter occasionally required adjustments when working with a new patient. The subsequent steps performed individual breath detection, lung ROI classification [14], and regional lung mechanics analysis [15]. We reasoned that this approach would be helpful in the intensive care unit, since very few parameter adjustments are required, and the variables quantifying lung mechanics are familiar to the clinician. Also, since our estimates of regional tidal volume are obtained from the average difference between images at end-expiration and end-inspiration, we do not anticipate a bias due to changes in pulmonary perfusion.

The EIT-based findings in our study of lung mechanics in ALI/ARDS are consistent with those from previous research using other modalities. First, lung recruitment studies using CT imaging have shown a heterogeneous distribution of lung density in ARDS [7], where the dependent lung regions are predominantly collapsed. The respiratory system compliance data in our study also suggest that the ALI/ARDS lung in our patients consisted of a patchy distribution of high and low compliance regions, each requiring different airway pressures for maximizing regional compliance. The patients in our sample also exhibited a trend of progressive lung recruitment in the ventral-to-dorsal direction, supporting the proposition that dorsal regions are subject to the gravitational force from ventral regions and, therefore, require additional pressure to inflate. Second, hysteresis of the ALI/ARDS lung has been previously studied by comparing static pressure–volume data acquired during inflation and deflation [24]. We found that the regional and total respiratory system compliance data also indicated the presence of lung volume hysteresis, where improved compliance was achieved during the PEEP Titration stage at lower pressures than those used during baseline and the lung recruitment stage. Finally, the correlations between changes in respiratory system compliance measured by EIT and by the ventilator suggest that the anatomical landmarks for electrode placement are appropriate for monitoring the regional lung mechanics in patients with ALI/ARDS. Although lung CT imaging is the gold standard for anatomical recruitment, such measures were unjustified in our study. The risks associated with the transportation of critically-ill patients out of the intensive care unit as well as those from ionizing radiation exposure during childhood were unacceptable.

**Table II:** Correlation Between EIT and Ventilator Compliance Data

*Patient*	*Steps*	*Correlation r*	*Significance p*
1	9	0.96	<0.01
2	14	0.94	<0.01
3	7	0.85	0.02
4	5	0.86	0.06
5	7	0.82	0.02
6	9	0.90	<0.01
7	7	0.94	<0.01
8	7	0.29	0.44
9	8	0.67	0.07
mean±SD	8±3	0.80 ±0.21	N/A

The proposed method, of course, has important limitations. First, the method is limited to detecting *reversible* atelectasis, since EIT image reconstruction is currently best-suited to measure impedance changes rather than absolute measurements. Consequently, only lung regions that exhibit some airflow during the ventilation protocol can be identified. We also assume that the transverse area occupied by the lungs does not change appreciably during recruitment. Although CT studies of lung recruitment support this assumption [7], [10], [17], its falsehood in a special case could affect the estimates of overdistension and atelectasis. Second, the processing steps following image reconstruction are relatively new and need further validation involving a broader range of patients with ALI/ARDS and different EIT systems. Making the programming sequence available to all centers is perhaps the most effective way to evaluate and optimize EIT-guided mechanical ventilation strategies for patients in respiratory distress. Although advanced alternatives to fEIT thresholding have been proposed for lung detection, e.g., least-squares regression [37] (supplement), [33], principal component analysis [38], and fuzzy logic pattern recognition [39], we reasoned that standard fEIT thresholding would serve as a good benchmark because its limitations are well understood, and it will challenge new methods remain as simple, automatic and fast as possible. The calculations for regional lung mechanics have only been demonstrated in two case studies of ARDS in adults [15] and, therefore, will also require further evaluation in both adults and children. Third, the method will systematically underestimate the “true” static respiratory system compliance, which is best obtained from the quasi-static pressure–volume curve, where pressure and volume measurements are made during the best practical approximation of zero flow [24]. Quasi-static pressure–volume maneuvers are unfortunately difficult to perform and require ventilator disconnect, making the risk-to-benefit ratio for ALI/ARDS patients unattractive.

It is clear from the epidemiological literature that a multicenter trial will be necessary to obtain a sufficient number patients to power a clinical trial of EIT-guided ventilation in ALI/ARDS. Therefore, the aforementioned challenges in data compatibility must be overcome before EIT-guided ventilation can be tested in terms of clinical outcomes, in particular, measures of morbidity and mortality. We recommend that the proposed approach be used as a framework for evaluating and improving each step of the sequence with the purpose of interactively guiding mechanical ventilation of the ALI/ARDS patient with EIT.

## Supplementary Material

Color versions of one or more of the figures in this paper are available online at http://ieeexplore.ieee.org.
